# Evaluation of Thermal Imaging System of the Scrotum in Patients with Varicocele

**Published:** 2017-12

**Authors:** Masoomeh DADPAY, Hossein GHAYOUMI ZADEH, Mostafa DANAEIAN, Farshad NAMDARI, Bijan REZAKHANIHA

**Affiliations:** 1. Dept. of Pathology, Faculty of Medicine, AJA University of Medical Sciences, Tehran, Iran; 2. Dept. of Electrical Engineering, Vali-e-Asr University of Rafsanjan, Rafsanjan, Iran; 3. Dept. of Urology, Faculty of Medicine, AJA University of Medical Sciences, Tehran, Iran

## Dear Editor-in-Chief

Varicocele is an abnormal dilation and tortuosity of the venous plexus above the testicles. It is a relatively uncommon condition before the age of 10 yr; however, its prevalence is 15% in young adults and 20% to 40% among infertile men (1). Currently, varicocele diagnosis and assessment depend on physical examination and scrotal ultrasonography/Doppler evaluation (2, 3).

One of the main explanatory theories regarding pathophysiology of varicocele is increased the temperature of the testicles (4). Thermal imaging is a remote, contactless, and non-invasive method. Elevated temperature at pampiniform venous plexus were reported higher than 34 °C or at testicles higher than 32 °C is indicative of testicular varicocele.

In the present study, thermography was performed using a VISIR 640 non-contact infrared camera. Its resolution was 110592 pixels per image and the minimum thermal resolution difference was 0.01 °C. SatIRWizard software was used for thermal analysis and reporting. Overall, 50 patients suspected of having varicocele were examined by a specialist clinician, and the medical diagnosis was recorded.

**Fig. 1:**
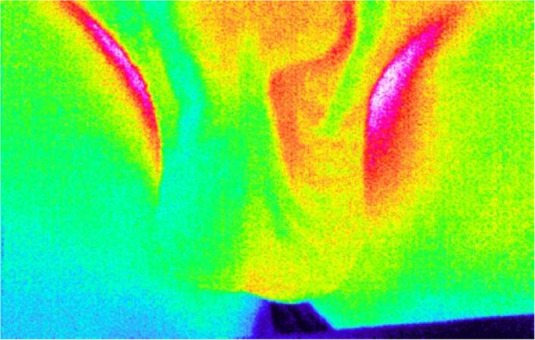
Asymmetric thermal pattern in the pampiniform venous plexus and the scrotum in thermography image

Several items including imaging condition, room temperature, patient convenience in the room, which could be effective on the results of the thermographic examination were evaluated before thermal imaging. The patients then took their clothes off and stood straight in front of the camera. They were asked to hold their penis up so that their legs were extended, their testicles were freely hanging between their legs, and the penis was held up against the abdominal wall. Finally, the operator prepared the thermographic image of patient’s testicles. Asymmetric temperature distribution pattern at pampiniform venous plexus can provide useful information regarding varicocele. An example of this pattern is depicted in Fig. 1.

Increased temperature is seen at both pampiniform venous plexus and scrotum.

The approximate temperature difference in patients with varicocele considering the database established using patients’ information and the grading system is provided in Table 1.

**Table 1:** Approximate temperature difference among patients with varicocele

***samples***	***Temperature differences in venous plexus Pampiniform***
Healthy case	ΔT<.5
Grade I	0.5<ΔT<0.75
Grade II	0.75<ΔT<1
Grade III	1<ΔT

The results obtained using ultrasound method was then compared with those of thermal imaging. The results of testicular ultrasonography in terms of grading system are presented in Table 2. In addition, the thermographic diagnostic functions were assessed considering patterns provided in this study. Five patients out of 50 evaluated cases were excluded due to their cystic lesion diameter.

**Table 2:** Comparison of thermography and ultrasonography results in terms of varicocele grading system

	***Grade No (%)***			***NO. (Healthy)***	***Total***
	I	II	III	-	-
Ultrasonography	8	12	10	15	45
Thermography detection	5	10	8	22	45

According to the results of this investigation and as thermography is a safe (patients are not exposured to radiation), inexpensive and relatively effective in varicocele diagnosis, it is considered a useful primary screening and complementary diagnostic method.
